# First results from the releases of Cinereous Vultures (*Aegypiusmonachus*) aiming at re-introducing the species in Bulgaria – the start of the establishment phase 2018–2022

**DOI:** 10.3897/BDJ.11.e100521

**Published:** 2023-03-09

**Authors:** Ivelin Ivanov, Emilian Stoynov, Georgi Stoyanov, Elena Kmetova–Biro, Jovan Andevski, Hristo Peshev, Simeon Marin, Julien Terraube, Lachezar Bonchev, Iliyan P. Stoev, Jose Tavares, Franziska Loercher, Marleen Huyghe, Zlatka Nikolova, Nadya Vangelova, Stamen Stanchev, Emanuil Mitrevichin, Elena Tilova, Atanas Grozdanov

**Affiliations:** 1 Green Balkans – www.greenbalkans.org, Stara Zagora, 9 Stara Planina Str., Bulgaria Green Balkans – www.greenbalkans.org Stara Zagora, 9 Stara Planina Str. Bulgaria; 2 Fund for Wild Flora & Fauna, 49 Ivan Mikhaylov Str., office 327, P.O.Box 78, www.fwff.org , pirin@fwff.org, Blagoevgrad, Bulgaria Fund for Wild Flora & Fauna, 49 Ivan Mikhaylov Str., office 327, P.O.Box 78, www.fwff.org , pirin@fwff.org Blagoevgrad Bulgaria; 3 Birds of Prey Protection Society, 23 Golyam Bratan Str., www.bpps.org, Sofia, Bulgaria Birds of Prey Protection Society, 23 Golyam Bratan Str., www.bpps.org Sofia Bulgaria; 4 Central European University, Department of Environmental Sciences and Policy, Vienna, Austria Central European University, Department of Environmental Sciences and Policy Vienna Austria; 5 Vulture Conservation Foundation, Wuhrstrasse 12 CH-8003 Zürich, Switzerland Vulture Conservation Foundation Wuhrstrasse 12 CH-8003 Zürich Switzerland; 6 South-West University „Neofit Rilski“, Faculty of Mathematics and Natural Sciences, Department of Geography, Ecology and Environmental Protection, Blagoevgrad, Bulgaria South-West University „Neofit Rilski“, Faculty of Mathematics and Natural Sciences, Department of Geography, Ecology and Environmental Protection Blagoevgrad Bulgaria; 7 Leuvensesteenweg 582, 2820, Muizen-Mechelen, Belgium Leuvensesteenweg 582, 2820 Muizen-Mechelen Belgium; 8 Department of Zoology and Anthropology, Faculty of Biology, Sofia University “St. Kliment Ohridski”, 8 Dragan Tsankov Blvd, Sofia, Bulgaria Department of Zoology and Anthropology, Faculty of Biology, Sofia University “St. Kliment Ohridski”, 8 Dragan Tsankov Blvd Sofia Bulgaria

**Keywords:** release from aviary, release by hacking (artificial nest), Balkan Mountains, survival rate, home-range, artificial nest platforms, Kotlenska Planina SPA, Sinite Kamani Nature Park, Vrachanski Balkan Nature Park, re-introduction strategy, evaluation in conservation management, raptor

## Abstract

The current work presents the preliminary results of the Cinereous Vulture (*Aegypiusmonachus*) releases in the Balkan Mountains in 2018–2022, aiming at the species re-introduction in Bulgaria, where it was listed as locally extinct since 1985. The first imports and releases of Cinereous Vultures in Bulgaria started in 2018. Until mid-2022, 72 individuals were released in the Eastern Balkan Mountains (Kotlenska Planina SPA and Sinite Kamani Nature Park) and Vrachanski Balkan Nature Park. Of them, 63 immatures imported from Spain were released from aviaries and nine juveniles captive-bred in European zoos were released by hacking (fledging from an artificial nest). We compared the success in survival and establishment between the different release sites and methods used to adjust the ongoing technics and tactics and to support knowledge improvement for future similar projects.

From the nine Cinereous Vultures released by hacking, the results were as follows: 1.00 fledging success, but only 0.22 survival in the first six months – combined period of acclimation, first migration and the first winter. All survivors from that period reached maturity into the wild, but all emigrated from the release site and settled elsewhere.

Of the 63 individuals released by aviaries, 32 individuals were released in the Eastern Balkan Mountains (18 individuals are still alive – 0.56 survival; 14 individuals settled in the area, which accounts for 0.44 of all released birds and 0.78 of the survivors). Thirty-one individuals were released in Vrachanski Balkan Nature Park (23 individuals are still alive – 0.74 survival; 22 individuals settled in the area – 0.71 of all released birds and 0.96 of the survivors). Based only on aviary method comparison, the settling of the individuals in the release area was alike in the two sites. However, the Vrachanski Balkan Nature Park performed better in survival – both in acclimation and establishment periods.

While comparing the release methods – hacking and release from the aviary – the following results were observed: the survival rate during acclimation was 0.86. Due to more considerable losses during the first migration and dispersal in the individuals released by hacking, the survival rate of 0.22 was significantly lower compared to 0.73 for the birds released from the aviary. Additionally, in both methods, a similar pattern in the first winter and spring migration dispersal was observed. Although the survival was equal in the released-by-hacking or aviary birds after the first year onwards, it is essential to note that the emigration of the hacked birds from the release site was 1.00. In comparison, the birds released from aviaries largely remained and settled in the release area (> 0.77 of the survivors). The cost of release and related acclimation, settling, dispersal and the first winter was the greatest: 0.12–0.17 per period, or cumulatively, it was about 0.27. Survival increased and stabilised to > 0.90 after the first year in the wild and reached nearly 1.00 after two years in the wild onwards.

Two distinct nuclei of the Cinereous Vulture were established along the Balkan Mountains – the Eastern Balkan Mountains with 18–23 individuals and four formed pairs using a territory of about 642.74 km^2^ – 95% home range and 85.72 km^2^ – 50% core area with center being the town of Kotel; and Vrachanski Balkan Nature Park with present 23–29 individuals, of which 2–3 pairs formed so far, using a territory of about 1,143.66 km^2^ – 95% home range and 22.89 km^2^ – 50% core area with center being the village of Zgorigrad. The species readily accepted breeding in artificial nest platforms built by professional arborists on different tree species – oak, beech, sycamore and pine. The only naturally built nests were on the ground (n = 2) (unsuccessful) and in Scots Pine (n = 1) (successful). In 2021 and 2022, in each of the two sites, the first successful reproductions were recorded, which marked the return of the Cinereous Vulture as breeding species – 28 years after the last occasional record of a single breeding pair in the country and 36 years after it was officially listed as locally extinct in Bulgaria.

## Introduction

The Cinereous Vulture *Aegypiusmonachus* – the largest bird of prey in Europe, classified globally as “near threatened” (BirdLife International 2021), used to be a widespread species in southern Europe. However, since the late 1800s and until mid-1900s, the species experienced a dramatic decline and disappeared from most of its range – including Bulgaria since 1985 (Patev 1950, Cramp and Simmons 1980, Botev and Peshev 1985, Hristov and Stoynov 2002, Stoynov et al. 2007, Marin et al. 2011). Remnant populations were still present in Spain, Greece, Ukraine (Crimea) and in the Caucasus, while since the 1990s, the species was re-introduced in the south of France. In Spain, from the 1980s to the present day, the species has been recovering, which has been possible thanks to the adoption of specific conservation measures, such as the ban and strict control on the use of poison baits, the protection of the main nesting colonies and the operation of feeding sites (Botha et al. 2017). In Greece, the Cinereous Vulture breeding colony closest to Bulgaria and the only surviving in the Balkan Peninsula is located in Dadia-Soufli-Lefkimi Forest National Park (hereafter Dadia) and has ranged between 21–35 pairs in the last three decades (Poirazidis et al. 2004, Skartsi et al. 2008, Botha et al. 2017). Despite the slow and slight increase in the number of pairs breeding in Dadia and, subsequently, the increase in observations of immature birds roaming across the border in Bulgaria (Arkumarev et al. 2020), still, no new breeding nucleus or colony has been identified neither in Bulgaria, Greece nor European Turkyie (Stoynov et al. 2019). Although a single pair breeding was recorded in 1993 in the Bulgarian part of the Eastern Rhodopes (Marin et al. 1998), this was an isolated event after about 20-30 years of no records and the pair disappeared after the breeding season, so the spontaneous return of the species in the country did not last.

Since 2010, local projects have been successfully implemented to re-introduce the Griffon Vulture (*Gypsfulvus*) at several places in Bulgaria: in the Eastern Balkan Mountains with two release sites (Kotlenska Planina SPA and Sinite Kamani Nature Park) (Kmetova–Biro et al. 2021), in Vrachanski Balkan Nature Park (Stoyanov et al. 2016), Central Balkan National Park (Stoev et al. 2016) and in the Kresna SPA (Peshev et al. 2019). The results were encouraging, with the establishment of 20–25 breeding pairs, 11 fledged young in 2016 (Stoynov et al. 2018b) and a continuing increase along the Balkan Mountains sites after more than half a century of absence from these areas. Using the Griffon Vulture local recovery in the target sites as proxy species, the initial results gave rise to the exploration and planning of conservation activities for the rarer Cinereous Vulture. A feasibility study was carried out to analyse the past and present state of the species in Bulgaria, to assess the ecological feasibility and to rate different sites for their ability to host colonies upon re-introduction (Stoynov et al. 2017, Stoynov et al. 2019). A National Action Plan (Kmetova-Biro et al. 2018) for the species was adopted by the Ministry of Environment and Waters of Bulgaria to restore the national population and re-establish some of the historical ranges, considering its importance also in a Balkan and European regional context. It framed the legal base and technically presented the process – import from Spain of Cinereous Vultures found in distress and rehabilitated, as well as captive-bred individuals from zoos, to be released in strategically-chosen sites throughout the country.

Considering that: 1. the presence of the Cinereous Vulture significantly decreased in the Balkans in the previous century as a result of multiple conservation threats and that it became nearly locally extinct; and 2. the last remaining nucleus in Dadia in Greece was relatively small and unable to expand in the neighbouring territories, re-introduction measures were recommended and justified by Stoynov et al. (2019) to restore the species in Bulgaria and to augment and increase the number and range of its population in the region. Two sites in Bulgaria – the Eastern Balkan Mountains (with two sub-sites – Kotlenska Planina SPA and Sinite Kamani Nature Park) and Vrachanski Balkan Nature Park – were selected to start re-introduction activities since they were rated with a higher score than the others at present and also because the number of individuals available to be released was limited. Know-how from projects in France and Spain was delivered by the Vulture Conservation Foundation (VCF) experts and translocated birds from Spain and captive-bred from European zoos (within the European Association of Zoos and Aquaria (EAZA) Ex-situ programme (EEP)) were planned to be released according to respective methods (see Material and Methods section).

The term “re-introduction,” as defined by IUCN (1987), is used to identify the intentional movement of an organism into a part of its native range from which it has disappeared or become extirpated in historical times. The re-introduction is considered successful when the species is “established,” meaning survival and successful breeding of both the founder individuals and their offspring is confirmed (Seddon et al. 2012, IUCN/SSC 2013). The “establishment phase of a re-introduction” refers to the period when the population is susceptible to particular threats that will disappear once the population survives this phase (Armstrong and Seddon 2008).

The stages and milestones in the establishment phase of the local re-introductions, foreseen in the feasibility study (Stoynov et al. 2019) and in general, were as follows:

1. Import of Cinereous Vultures for release in Bulgaria;
2. Establishment of local non-breeding nuclei – more than a single bird and permanent presence throughout the year in the target release sites;
3. Post-release effects (Armstrong and Reynolds 2012) no longer play a role over the bulk of the released birds and the first successful breeding by founder individuals is recorded;
4. The establishment phase of the Cinereous Vulture local re-introduction will be considered complete once the local breeding nucleus has started producing about ten offspring a year and locally fledged individuals have started reproducing on their own – expected as a mid-term achievement (by 2030).

Local re-introductions of the Cinereous Vulture have previously been conducted in Grand Causses, Verdon and Baronies in France (Mihoub et al. 2013b, Rousteau et al. 2022), as well as in Catalonia and Burgos in Spain (Andevski et al. 2017). In these projects, data for whereabouts, adaptation and survival was, until recently, obtained by plastic ring recoveries, by direct sight or photographing the re-introduced individuals. On some occasions, GPS transmitters were used. In the current study, all re-introduced birds were intensively GPS-tracked since their release and their survival, adaptation, feeding, movements and migrations, roosting, breeding and territory use was documented in detail, all of which were highly recommended for re-introduction programmes (Seddon et al. 2007, Sutherland et al. 2010, Efrat et al. 2022).

According to Collier et al. (2018), while also citing the respective authors, *acclimation* is defined as the coordinated phenotypic response developed by the animal to a specific stressor in the environment (Fregley 1996), while *acclimatisation* refers to a coordinated response to several simultaneous stressors (e.g., temperature, humidity, and photoperiod (Bligh 1976). Acclimation and acclimatisation are induced by the environment and are considered phenotypic and not genotypic change, and the responses decay if the stress is removed. Acclimation and acclimatisation act to improve animal fitness to the environment. The term "acclimatisation" in the current study is used for the period the birds spend in the aviary to get used to the environmental specifics of the site and socialise with the group of conspecifics before release. The aviary is, thus, called an "acclimatisation aviary" (Terrasse et al. 2004). The term "acclimation" is used here to describe and evaluate the success of releases in the very first weeks and months in the wild (in line with Parker et al. 2012). It is similar to the abovementioned term but refers to the period after the release when the birds are disorientated and recover their flight abilities, discovering roosting, feeding and watering sites or learning to differentiate between potentially life-threatening and benign situations (Levine and Ursin 1991). This division is essential from a conservation management perspective because the "acclimatisation" reflects more the rearing of the birds in captivity (in the aviary before the release), the "acclimation" reflects the release and post-release care success and the establishment success is related to these two, but also to the habitat quality. Therefore, all these aspects must be evaluated to inform the ongoing activities and other reintroduction projects.

This paper provides data and discusses details on the releases, acclimation, observed post-release effect, survival and the preliminary results from the ongoing establishment phase – breeding, mortality, migration, territory occupancy and use (home-range) from the first five years (2018–2022) of the re-introduction of the Cinereous Vulture in Bulgaria, some of which have direct implications for the species conservation and release methods adjustment.

## Material and Methods

### Release and management techniques

**Figure 1.**
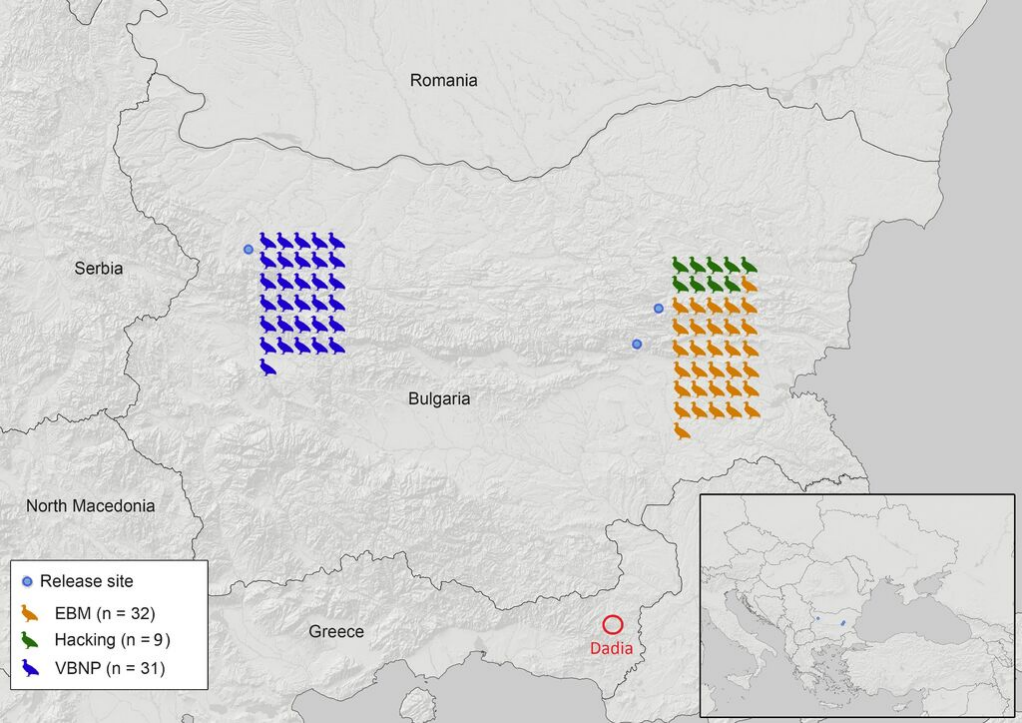
A map of the location of the Cinereous Vulture release sites in Bulgaria and the respective number of individuals released by site and method in the period 2018–2022. The red circle with the inscription "Dadia" points out the location of the last autochthonous colony of the Cinereous Vulture in the Balkan Peninsula – Dadia-Lefkimi-Soufli Forest National Park.

In the period 2018–2022, a total of 72 Cinereous Vultures (detailed data provided in Suppl. material 1) were released at two sites in Bulgaria (see Table 1): 1. the Eastern Balkan Mountains (hereafter EBM) with two sub-sites – Sinite Kamani Nature Park (UTM, MH43) and Kotlenska Planina SPA (UTM, MH65) – due to the proximity of the two release sites (< 20 km line of sight) and the common movement patterns and behaviour of the vultures released so far, the two sites were reviewed as a single site; and 2. Vrachanski Balkan Nature Park (hereafter VBNP) (UTM, FN99). Two different methods were used to release the vultures: 1. captive-bred juveniles fledged from an artificial nest, known as hacking (Sherrod et al. 1982) and 2. translocated immatures released through an acclimatisation aviary (Terrasse et al. 2004) (see Fig. 1). Nine captive-bred juvenile Cinereous Vultures provided by European zoos (through the EAZA EEP) in their 90^th^ day after hatching, were released in EBM by hacking in 2018 (n = 3), 2019 (n = 4) and 2022 (n = 2), while 63 translocated immature individuals from Spain were released in spring/summer after spending 3–9 months in an acclimatisation aviary on the respective release sites – 32 individuals in EBM in 2019, 2021 and 2022 and 31 individuals in VBNP in 2020, 2021 and 2022.

Acclimatisation aviaries and feeding sites (also known as "supplementary feeding sites" or "vulture restaurants", Piper et al. (2009)) were built on the three abovementioned release (sub)sites in the previous project for the re-introduction of the Griffon Vulture (Stoynov et al. 2018b). The same structures were employed for the releases of the Cinereous Vultures. Following the Griffon Vulture and later-on Cinereous Vulture release and adaptation methodology described by Terrasse et al. (2004), food was provided in the long-term (since 2010) and permanently supplied at fenced feeding sites close to the hacking platforms (built nearby in purpose) and in front of the acclimatisation aviaries. On average, 170–220 provisions a year (2–4 times a week) with 30–60 tonnes per site (50–500 kg per provision) were provided.

Artificial nesting platforms were built for the Cinereous Vultures, based on the information from published habitat and nest-site selection models (Poirazidis et al. 2004, Morán-López et al. 2006, Yamaç 2007, Mihoub et al. 2013a, Guerrero-Casado et al. 2013, Ortiz-Urbina et al. 2020). The nesting platforms were built by professional arborists and, in some cases, only the crowns of the trees were shaped to provide horizontal branches and a cup on the top. Different species of trees were used for this purpose: oak, beech, sycamore and pine. The trees selected were at a distance between 0.5 and 10 km from the release sites. The main factor for choosing the right spot and trees was the repeated visits and overnight roosting by the GPS-tagged pairs in the particular area.

### Monitoring techniques

For the purpose of monitoring and analysis, the borders of the release area had to be defined. The term "release area", therefore, refers to the two Natura 2000 sites – Kotlenska Planina SPA (BG0002029) and Sinite Kamani-Grebenets SPA (BG0002058) (comprising the Sinite Kamani Nature Park) and the territory between them in EBM, covering a total area of 2,826 km^2^ – a circle with 30 km radius with the centre being the town of Kotel (in line with Stoynov et al. 2017). The VBNP release site covers (for the study) Vrachanski Balkan SPA (BG0002053), parts of Ponor SPA (BG0002005) and Zapaden Balkan SPA (BG0002002), a total area of 2,826 km^2^ – a circle with 30 km radius with the centre being the village of Zgorigrad. The release sites were subject to intensive conservation and management measures aiming at reducing threats (e.g. insulation of power lines, prevention of poisoning, raising public awareness in conservation) and improving the habitat quality (e.g. intensive food provision and feeding sites maintenance, artificial nest platforms building, support of extensive livestock breeding) for the locally-re-introduced vultures.

Тo ensure individual identification of the Cinereous Vultures that were released within the re-introduction project, all were marked with standard metal ornithological rings, PVC colour rings and GPS/GSM transmitters. In addition, the first two chicks hatched into the wild were also marked with rings and GPS transmitters before fledging to follow their dispersal and survival.

Out of collecting and analysing GPS data from the transmitters, the vultures visiting the three feeding sites and the identified roosting sites were monitored weekly through direct observations and recording of the individual birds present. Additionally, camera traps were also used at the feeding sites and the footage was analysed regularly, recording the rings of the birds identified, as well as the maximum number of vultures counted (both tagged and non-tagged) on site. Finally, all observations were manually entered into an online storage database with some analytical functions.

**Figure 2.**
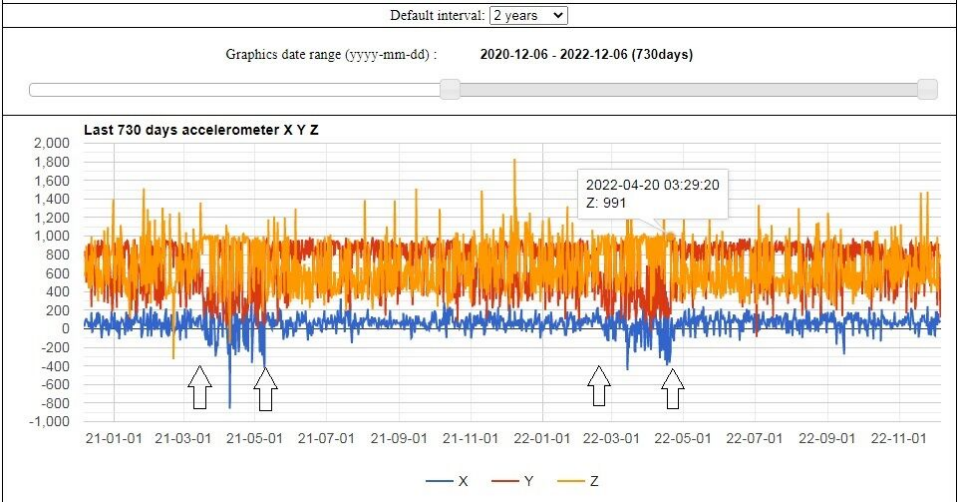
Accelerometer data graph from two years of GPS tracking of the Cinereous Vulture called "VCF Know-how" that was involved in incubation during two consecutive breeding cycles. The black arrows indicate the start and end (left to the right) of the periods of temporal disruption of the "normal" accelerometer data graph pattern, which coincides with the incubation period when the bird is frequently lying in the nest.

Breeding attempts were recorded, based on GPS data – disruption of the accelerometer's data graphics pattern (see Fig. 2), but also observations were carried out in good weather and visibility, at a distance of 500 to 1300 m from the particular nest to minimise disturbance, using spotting scopes (30×60 and 20–60×80). For all identified nests, the following information was reported:

- breeding birds (identified in the majority of cases through their GPS tags and colour rings), nest location coordinates;
- time of occupation;
- activity at the time of the observation (nest building, mating, lying/incubation, chick rearing and independence).

In addition, in order to follow the yearly progress and development of the population demography, the following data were collected:

1. the number of the individuals present in the release area;
2. the number of territorial pairs;
3. the number of breeding pairs (pairs that were observed incubating);
4. the breeding success (fledged juveniles per incubating pair);
5. the productivity (fledged juveniles per occupied nest);
6. the number of occupied nests (all nests occupied by breeding and non-breeding pairs) and the substrate used, as well as if the occupied nest was an artificial platform or naturally built by the birds.

The GPS/GSM transmitters (produced by Ornitela UAB - www.ornitela.com) weighed from 30 to 50 g or < 1% of the body mass of the birds tracked – following the recommendation of < 3% for flying birds (Kenward 2001). The devices were attached to the birds' lower back by a leg-loop harness (OT-30 and OT-50), prepared by three assembled strings (round silicone cord 2 mm + tubular Teflon ribbon 0.25" and 0.44") according to VCF's internal rules (Daniel Hegglin and Franziska Lörcher – pers. comm.). In order to guarantee that the device would fall off in a couple of years, a vulnerable attaching element was deliberately used while fitting. Furthermore, the transmitters were mounted following the best practice in animal welfare – the heads of the birds were covered to ensure minimal stress and the transmitter placement time was reduced to less than ten minutes.

All Cinereous Vultures locations were obtained using a global positioning system (GPS), transmitted via a public mobile phone/internet system network (GSM/GPRS). The devices were programmed to save the location data if birds were outside of the coverage area of the given network operator and then to send it once the transmitter was back within range. GPS fixes were acquired every 10 min during the day (between 0500 h and 2000 h UTC+2) with dormancy periods during the night and to send the data every 1-6 hours, depending on the battery charge.

### Calculations and statistics

Breeding success, the number of fledglings, survival rate and demographic parameters were calculated based on annual averages. Mortality cases were grouped by cause and provisioned as an absolute number for comparison. Additionally, the drop-out of the released individuals was grouped by the time after release and buffer periods were set up as follows: 0–6 months – acclimation and first autumn migration/dispersal - all released birds were intensively monitored through GPS transmitters and subsequent visits to the field were conducted when GPS data showed the absence of movement suggesting death or tag-failure. Given this, casualties could be clearly separated from cases of transmitter-failure and the estimates reported in this paper correspond to true survival; 6–12 months – first winter and first spring migration/dispersal; and > 12 months. This approach is in line with Sarrazin et al. (1996) and Sarrazin (2008) to analyse whether recapture/death has occurred as a post-release effect as a possible result of a lack of personal experience in the wild (Tavecchia et al. 2009) or sub-optimal habitat selection (Mihoub et al. 2009). The survival of the individuals was calculated to specific periods – for example, a) up to 2 months after the release (acclimation); b) up to 6 months (settling); c) up to 1 year, which will coincide with the differences in the adaptation – post-release effect, adaptation/settling effect and managing to survive through extreme winter conditions (the first winter) and then survival to second, third and following years to assess the suitability of the sites/habitats, later adding also the reproductive success. Relative survival was calculated as the number of survivors divided by the number of live individuals from the preceding period. The results were then compared to the post-release and natural mortality reported for other re-introduction projects and wild populations, such as those published by Rousteau et al. (2022) for France.

Prior to analysis, the tracking data were inspected and visualised in the Quantum GIS free and open-source, cross-platform desktop geographic information system (QGIS.org 2021) to check for outliers and all duplicate coordinates were removed. Only locations taken between 0600 h and 1800 h UTC+2 within the borders of the Balkan Peninsula were used to determine the home ranges, while the rest of the coordinates in the studied hourly range were used for establishing the roosting sites. The location error was less than 20 m.

The local home ranges of the Cinereous Vulture were calculated on the basis of a total of 4,384 tracking days of four tagged vultures in EBM and 3,327 tracking days of four tagged vultures in VBNP. A dataset, comprising about 322,000 GPS fixes, was analysed (Table 2). The home range of each vulture was calculated using the dynamic Brownian Bridge Movement Model (dBBMM) (Kranstauber et al. 2012). Statistics were undertaken with the R free software environment for statistical computing and graphics Version 4.0.3 (R Core Team 2020), using the "adehabitatHR" (v.0.4.18; Calenge (2006), Calenge (2019)) and the "Move" (v.4.0.6; Kranstauber (2020)) packages. The 95% utiliity distribution (UD) was defined as the general individual home range and the 50% UD was defined as the core area.

## Results and Discussion

Of the 72 individuals released, nine were released by hacking (artificial nest), out of which only two survived the acclimation period and reached maturity. Eventually, they settled in Çankiri in Turkiye (n = 1) and Dadia in Greece (n = 1) and the latter bred unsuccessfully in 2022. The fledging success of the hacked Cinereous Vulture chicks was high – 1.00. However, only 0.22 (SD = 0.31) of them survived the first six months – the combined acclimation period, the first migration and the first winter. All the birds surviving this period also reached maturity into the wild, but they all permanently emigrated from the release site (see Table 3).

Sixty-three immature Cinereous Vultures were released from aviaries. Of them, 52 individuals (0.83) survived the acclimation period (first six months after release), none died during the first autumn migration/dispersal, six died the first winter and two died in the first spring migration/dispersal. The four periods lasted between 6 and 12 months following the release and comprise a total survival of 0.73 (SD = 0.17). Forty-one Cinereous Vultures (0.65) were alive at the time of reporting – seven individuals survived more than 48 months in the wild (accounting for 0.64 survival of their batch – the first one released in EBM); six individuals survived more than 24 months in the wild (accounting for 0.60 survival of their batch – the first one released in VBNP); and thirteen individuals survived more than 12 months (accounting for 0.57 survival – the second batches released in EBM and VBNP). The batches were released in different years. The survivors from the last two batches - 18 individuals released in spring/summer 2022 (EBM n = 7 and VBNP n = 11) just passed their six months period in the wild and were entering their first winter season at the time of reporting, but, meanwhile, two died depredated by the Golden Jackal (*Canisaureus*) in VBNP. The release and related acclimation, settling, dispersal phases and the winter during the first year into the wild were particularly costly in terms of the loss of released individuals – from 0.12 to 0.17 per period or a cumulative release cost of about 0.27. Survival increased and stabilised to around 0.90 after the first year in the wild and reached nearly 1.00 after two years in the wild onwards. Sarrazin and Legendre (2008) proposed releasing adult Griffon Vultures instead of juveniles to avoid emigration and related losses in re-introductions. However, Rousteau et al. (2022) stated that there were no differences in the survival of the Cinereous Vultures from the two age groups. The local conditions in the Balkan Mountains at the chosen release sites may be encouraging the hacked juveniles to migrate soon after fledging. Autumn migration was not observed in the two locally reared chicks in EBM in 2021 and VBNP in 2022, both which remained in their natal area during the first winter. Out of the cost of release, the EBM also showed higher mortality in the post-acclimation period. This is probably related to stochastic events, more frequent emigration or Allee effects (e.g. Courchamp et al. (2008)) on the newly-established nucleus, as, after the release in 2019, the next 2020 was a gap year, which was not the case in VBNP. Eventually, this might provide differences in social interactions and related loss of individuals that did not find mates due to emigration.

Out of the survival rate between the different batches in EBM, there was a difference in the survival rate and related settling success between the two release sites – EBM and VBNP as follows:

- 32 individuals were released in EBM (18 individuals are still alive – 0.58 (SD = 0.27) survival; 14 individuals settled in the area, which accounts for 0.44 of all released birds and 0.78 of the survivors).
- 31 individuals were released in VBNP (23 individuals are still alive – 0.74 (SD = 0.10) survival; 22 individuals settled in the release area beyond the acclimation period accounting for 0.71 of all released birds and 0.96 of the survivors);

The VBNP release area performed better, based on higher survival during the acclimation period than in the EBM, where jackals predated several individuals in the first days following the release in Sinite Kamani Nature Park (n = 4). Meanwhile, just during the preparation of the recent manuscript, four Cinereous Vultures (also the first fledged chick in the wild in VBNP in 2022) were also depredated by jackals in VBNP in their first winter period (December 2022). There were fewer losses and emigration amongst the settled individuals in VBNP compared to EBM. The latter site faced a poisoning incident in March 2022, which killed four individuals at once (including the first wild-fledged chick in the area). Several others died for different reasons – electrocution/collision and storm-crashed tree fall. The EBM also lost birds during dispersal phases away from the release site: shooting (n = 3), poisoning (n = 1) and drowning in sea or water reservoirs (n = 3). The mortality cases by number and cause are summarised in Table 4.

Comparing the release methods – hacking and release from an aviary, the mean survival rate during acclimation was the same – 0.86, as was also reported by Rousteau et al. (2022). However, more considerable losses during the first migration and dispersal of individuals released by **hacking** were observed in EBM and, thus, a difference in the survival rate – 0.22 compared to 0.73 of the birds released by an aviary. This difference could be attributed to the overall lower probability of migration and dispersal of the birds released by an aviary (due to their age > 1 year old and related to the initiation of mate searching and territorial settlement that fixes them). Additionally, in both methods, a similar pattern in the first winter and spring migration dispersal was observed. Although the survival was equal in the birds released by hacking or by an aviary after the first year onwards, it is essential to note that the emigration of the hacked birds from the release site was 100%, whereas the birds released by aviaries largely remained and settled in the release area (> 0.77 from the survivors). The remaining 0.23 of the birds released by aviary that emigrated from the release area settled in the other project site – one bird from EBM went to VBNP and conversely. Interestingly, both birds settled in the other project area were females attracted by locally-settled males, which suggests that the males establish territories and then attract females there. The sample is too small, but this question should be further studied, as this might have implications for creating local population nuclei and for re-introduction success.

The fate of the released Cinereous Vultures in the Balkan Mountains release-sites in Bulgaria in 2018–2022 is summarised in Table 5. Electrocution was locally considered the most critical threatening factor based on the knowledge of the releases of the Griffon Vulture (Kmetova-Biro et al. 2018). Poisoning is the top threatening factor for vultures globally and in Europe (Botha et al. 2017). This was the reason for considering these factors as top anthropogenic threats for the Cinereous Vulture in Bulgaria. It appeared, however, that electrocution is a less critical threat to the local Cinereous Vulture's population. This is because they prefer landing on the ground and very rarely perch on pylons (mainly when there is a snow cover and no cliffs or suitable trees nearby). The poisoning is a serious threat, but more for settled individuals than for the newly-released ones. Thus, the factor is not essential for acclimation, but indeed for the establishment and long-term survival of a nucleus/population. With 0.31, the depredation of Cinereous Vultures in their acclimation period and the first winter was the most severe threat with the highest cost of release effect. The shooting - 0.17 of all mortality cases is the most severe anthropogenic threat in the acclimation period. It has happened in various locations – close or far to release sites (e.g. S and SW Bulgaria and Hungary). The behaviour of the birds (landing on the ground and sitting for hours) provoked the interest of the shooters. The same behaviour in the very first days after the release in the very core of the release areas became the reason for depredation by jackals of newly-released birds in EBM, but a very recent event from December 2022 also involved the first fledged offspring in the VBNP and three more birds (6 to 18 months after release) roosting on the ground during the night. Although the large predators experienced a comeback in Europe in the last decades (Chapron et al. 2014), depredation of Cinereous Vultures was never reported from other re-introduction projects, which took place only in western Europe. Stoynov et al. (2018a) stressed that the number of wolves (*Canislupus*) in the Balkans is two times higher and the range is five-fold more extensive than in the Iberian Peninsula, where the vultures have thrived in the last decades. The authors also noticed that wolves and vultures rarely exist sympatrically in Europe, mainly due to the use of poison by shepherds in revenge for the predation of livestock by wolves. This might be a clue to the difference in abundance of the vultures in the Balkans and the Iberian Peninsula despite similarities in size, climate and other features. Additionally, the Golden Jackal is not present in western Europe. At the same time, the species' population in Bulgaria has faced a 9-fold increase from 5,000 individuals in the 1990s to 47,774 individuals in 2015 (Vlaseva 2018). Consequently, in our study area, Cinereous Vultures roosting on the ground and the abundance of medium and large terrestrial predators bring a new problem - loss of individuals due to depredation - entirely unexpected for one of the largest birds of prey. Another case of Cinereous Vulture suspected depredation by jackals has been recently reported from Iran for an immature individual from the EBM 18 months after release. This threat is very unusual and should be addressed by adaptive management in re-introduction approaches (McCarthy et al. 2012), based on local circumstances monitoring and proactive initiatives by the re-introduction managers.

Shooting, poisoning and electrocution, the three top threats for the vultures globally (Botha et al. 2017), formed 0.44 of the threats. These threats should be addressed on a large scale, but especially intensively in the Vulture Safe Areas (Peshev et al. 2021), as the chosen release sites for re-introduction programmes should be devoid of important anthropogenic threats.

Drowning into the sea happened to one hacked juvenile on its first autumn migration and one immature one year after the release. This problem is the one described by Buechley et al. (2021) for the juvenile Egyptian Vultures (*Neophronpercnopterus*) from the Balkans during their first autumn migration. A third immature bird drowned in a muddy substrate in a reservoir in Ukraine. This factor is essential and should be considered in re-introduction programmes, yet it is impossible to control. Therefore, it should be put in the occasional deaths list along with the natural disasters and diseases, for which the total weight in the current study provides 0.30 of all mortality factors. However, drowning in the sea is a consequence of long migrations and dispersal, which could be minimised in re-introduction programmes (e.g. avoiding releases of juveniles applying the so-called 'delayed release' method; Arkumarev et al. (2021)).

The first breeding behaviour and pair formation was observed in 2020 – a year after releasing the first batch of immature birds from the aviary in EBM. Three territorial pairs were formed by 3-year-old birds (hatched in 2017). The birds from the formed pairs stayed and usually overnighted together, away from other conspecifics, as seen by the GPS transmitters data. The places that the territorial pairs frequently used for overnight roosting were recognised as potential breeding sites and artificial nest platforms were established. The first nest occupations and courtship behaviour were observed in 2021 when the involved birds were four years old. The species readily accept breeding in artificial nest platforms built by professional arborists in different kinds of trees: oak, beech, sycamore and pine. The only naturally-built nests were on the ground (n = 2) (unsuccessful) and in a Scots Pine (*Pinussylvestris*) (n = 1) (successful). Table 6 summarises the results. The observed low breeding success (0.33) may be attributed to the young age of the pioneer pairs that started reproducing so far. More time is needed to evaluate if the young age of breeding individuals or suboptimal habitat (e.g. disturbance or lack of suitable nesting trees; Morán-López et al. (2006), Kirazlı (2016)) may be the reason for the observed breeding performance. The readiness of the released Cinereous Vultures to occupy artificial nest platforms in different tree species (e.g. decidious oak, beech and even sycamore) is promising, as almost all case studies show a preference for nesting on pines, for example, in Greece (Poirazidis et al. 2004), Turkyie (Yamaç 2007), France (Mihoub et al. 2009) and Spain (Ortiz-Urbina et al. 2020) and less often on evergreen oaks and juniper for example Spain (Morán-López et al. 2006, Moreno-Opo et al. 2012). Thus, if proven successful in the long-term, if this were not an Allee effects performance or like, the species would repopulate much of the historical range, using habitat with continental climate vegetation, even beyond the Mediterranean climate range, as much as it is related to the nesting habitat features. This new finding might be important in terms of the Cinereous Vulture's adaptation to changing climate and the related increase in forest fires in the drier environments – threatening the top preferred breeding habitats (according to Gavashelishvili et al. (2006)) for the species.

The pairs formed when the birds were immature, about two years old and stayed together and usually kept away from other conspecifics. Change of mates was observed in five pairs – one male (from EBM) lost its female (also from EBM), which died from a tree crash in a storm and soon replaced it with another female (from VBNP), which was by then part of another pair (a male also from VBNP). The left-alone male returned to VBNP and took a new local female; two pairs in EBM – once established, after some time permanently swung their partners; in one pair – although successfully bred in 2021, the male has been chased away and replaced by another (still immature) male, while the latest usurped the female and the nest. The breeding season started with nest building and preparation in the summer preceding the first nesting. The nesting period stages are summarised in Table 7.

**Figure 3.**
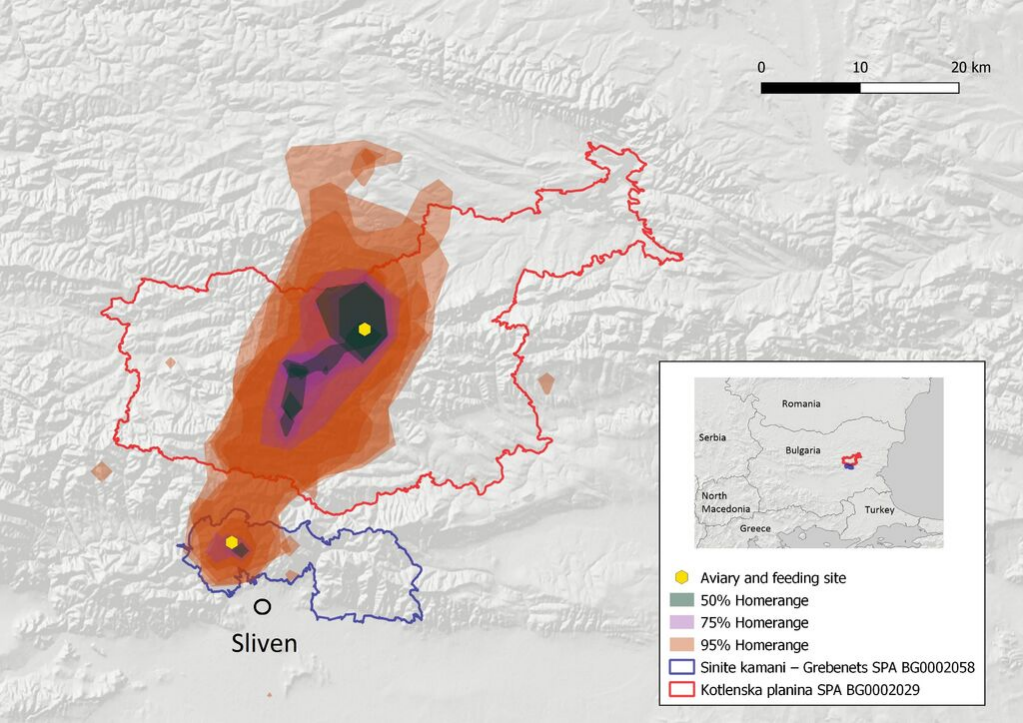
Map of the Eastern Balkan Mountains release sites and home ranges of the newly-established Cinereous Vulture nucleus.

**Figure 4.**
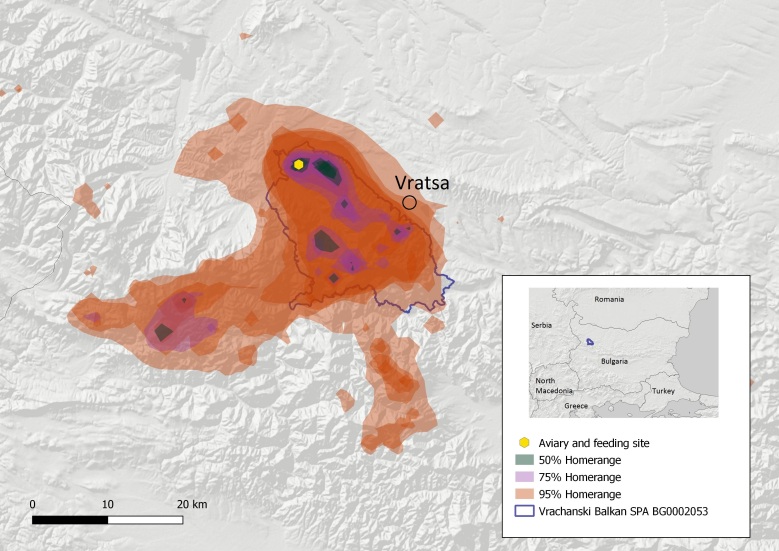
Map of the Vrachanski Balkan Nature Park and adjacent Natura 2000 sites and the home range of the newly-established Cinereous Vulture nucleus.

**Figure 5.**
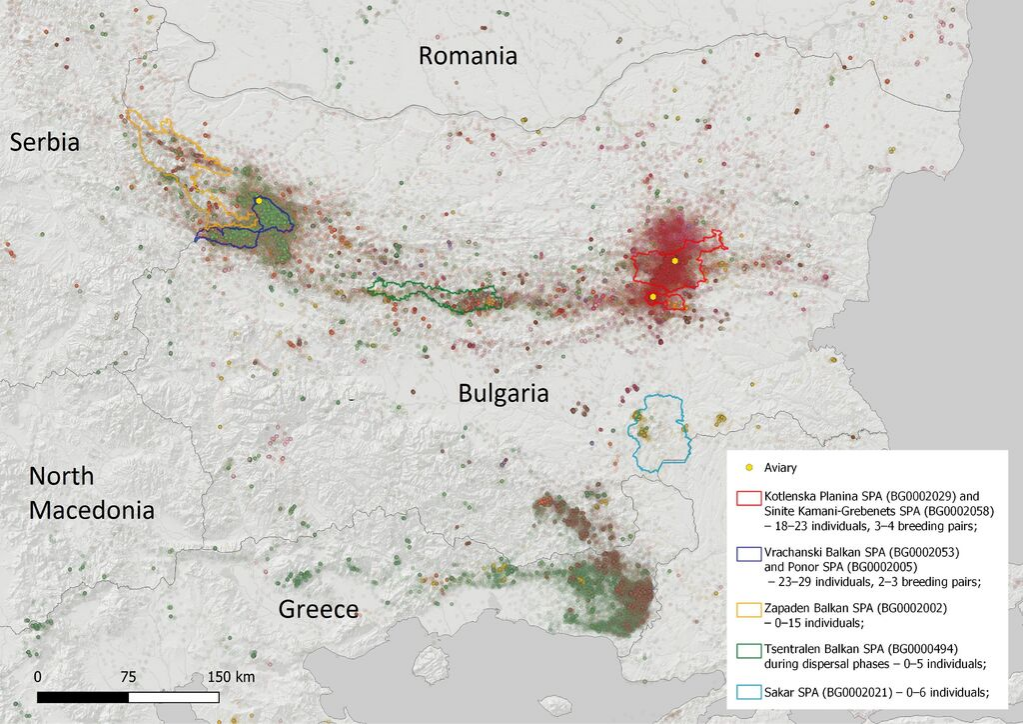
Map of the movements and sojourn of the GPS-tracked Cinereous Vultures in Bulgaria and adjacent countries with emphasis on the presence in certain Natura 2000 zones.

The two distinct nuclei of the Cinereous Vulture were established along the Balkan Mountain: 1. The EBM with 18–23 individuals (including exogenous birds) and four formed pairs were using the territory of about 642.74 km^2^ – 95% home range and 85.72 km^2^ – 50% core area with a centre being the town of Kotel (Fig. 3); and 2. the VBNP with presently 23–29 individuals (including exogenous birds), of which 2–3 pairs have been formed so far and are using the territory of about 1,143.66 km^2^ – 95% home range and 22.89 km^2^ – 50% core area with centre being the village of Zgorigrad (Fig. 4). The calculated home ranges are smaller compared to France - the average 95% home range size of the Cinereous Vultures in France is maximal in summer (4,713.17 ± 2,886.05 km^2^) and lower in winter (1,935.59 ± 2,013.14 km^2^, Rousteau (2020)). However, these values in France are much higher than the estimates from Greece (540 km^2^ on average, Vasilakis et al. (2008)) or in Spain (1,354.30 km^2^ on average during the breeding season and 777.75 km^2^ in the non-breeding season, Carrete and Donázar (2005)). The home range of the Cinereous Vulture in EBM is similar compared to Greece and the one in VBNP is similar to that in Spain. It is early to define the home range of the two newly-established nuclei, as they will probably develop further in the post-establishment period. At this stage, it is essential to provide preliminary data and show the current territory use to direct precise conservation measures and ensure legal protection by actualising the Standard Data Forms of the respective Natura 2000 zones (see Fig. 5).

## Conclusions

The Cinereous Vulture re-introduction establishment phase in Bulgaria in the two first release sites in EBM and VBNP is running according to the plan and the first results are satisfactory. Two distinct nuclei are now created and the species started breeding, which might be a reason to up-list it in the Red Data Book of Bulgaria from "Extinct" to "Critically Endangered." Additionally, the figures in the Natura 2000 Standard data forms in the respective sites should be updated as follows: Vrachanski Balkan SPA (BG0002053) and Ponor SPA (BG0002005) – 23–29 individuals, 2–3 breeding pairs; Kotlenska Planina SPA (BG0002029) and Sinite Kamani-Grebenets SPA (BG0002058) – 18–23 individuals, 3–4 breeding pairs; also the species should be listed as present in Tsentralen Balkan SPA (BG0000494) during dispersal phases – 0–5 individuals; Zapaden Balkan SPA (BG0002002) – 0–15 individuals; and Sakar SPA (BG0002021) – 0–6 individuals. For the first time, specific data for breeding cycle dates of the Cinereous Vulture in Bulgaria are available. The establishment phase of the re-introduction of the Cinereous Vulture has started and is now ongoing. Further monitoring and modelling for the long-term persistence should be provided and, as much as necessary, adaptive management of the new national population of the species should be applied.

The presented data show that the hacking method is inefficient for establishing a nucleus of Cinereous Vultures from zero in the Balkan Mountains (given the actual circumstances) in Bulgaria, nor for supplementing a small settled group of individuals. Alternatively, the method should be further tested with, examined or experimentally replaced with the aviary method - releasing the captive-bred birds after a certain period of acclimatisation and gaining life experience (delayed release) to increase the success in survival and settlement in the project's Vulture Safe Areas.

## Acknowledgements

Since 2014, the Cinereous Vulture re-introduction in the two release sites along the Balkan Mountains of Bulgaria was implemented by three Bulgarian NGOs - namely Green Balkans, Fund for Wild Flora and Fauna and Birds of Prey Protection Society. The project "Vultures Back to LIFE" - LIFE14NAT/BG/649, in which partners are also the Vulture Conservation Foundation, EuroNatur and Junta de Extremadura, has been co-financed by the LIFE+ financial instrument of the European Commission.

The Vulture Conservation Foundation, besides this paper's co-authors, Hans Frey, Alex Llopis and Raphael Neouze, provided the methodology, know-how transfer, data collection tips and analysis.

The activities of the Green Balkans in Sinite Kamani - Grebenets SPA and the data collection have also been co-financed by the Stichting Wildlife, Netherlands, Jeremie Touchard, Sinite Kamani Nature Park Directorate, DierenPark Amersfoort, the Netherlands.

The Fund for Wild Flora & Fauna's activities and data collection in Kotlenska Planina SPA and Kresna SPA have also been co-financed by the Bioparc Conservation, France, Sainte Croix Biodiversite, France, Görlitz Tiergarten, Germany and Mulhouse Zoo, France.

The activities of the Birds of Prey Protection Society in Vrachanski Balkan were backed by Vrachanski Balkan Nature Park Directorate, but also by Green Balkans and FWFF and their contributors.

We are incredibly grateful to:

- the Vulture Conservation Foundation, AMUS, Los Hornos and the Spanish Government, as well as the European Association of Zoos and Aquaria's Ex-situ Programme (EEP) and the European zoos (Zlin Zoo, Ostrava Zoo, Riga Zoo, Planckendael Zoo, Parc des Oiseaux, Prague Zoo, Liberec Zoo,), who have provided Cinereous Vultures for release, as well their expertise and experience in vulture release, data collection and processing.

- also to 1. the Wildlife Rehabilitation and Breeding Centre of Green Balkans – Dr Rusko Petrov, Dr Stefka Dimitrova, Andreana Dicheva and all other team members in Stara Zagora in Bulgaria and 2. Maria Ganoti and the ANIMA - First Aid Station team for wildlife in Greece for necropsies, sampling and investigating Cinereous Vultures' mortality cases in the respective countries.

- to Miroslav Enev and his team of arborists for nest building, data collection and marking the chicks in the nests and to Lybomir Andreev for video and photographic material provided, which was used for additional information gathering for the Cinereous Vulture.

- to many vulture enthusiasts and experts who provided data for Cinereous Vultures observations and recoveries from their areas/countries: Dr Rigas Tsiakiris, Cornel Cotrogea, Sylvia Zakak, Elzbieta Kret, Dr Volen Arkumarev, Marin Kutrtev, Giannis Psarakis, Burak Tatar, Tamer Yilmaz, Mehmed Kiran, Tora Benzeyen, Zahra Elahi Rad, Theodora Skartsi, Dimitris Vavylis, Dimitris Vasilakis, Marton Horvath, Árvay Márton, Deák Gábor, Juhász Tibor, Nenad Petrovski, Emanuel Lisichanets, Ferdinand Liner, Tomislav Bandera Anić, Svilen Cheshmedzhiev, Yordan Kutsarov, Dionysis Mamasis, Nicolaos Nulas, Lőrinc Bărbos, Gergő Halmos, Emanuel Baltag, Lucian-Eugen Bolboaca, Oana Vasiliu, Viorel Gavril, Silvia Ursul, Alexandr Turkan, Vitalie Ajder, Maxim Yakovlev, Oleg Sheremet, Oleg Sapuga, Vadim Rudenko, Svilen Stamatov, ACDB-Action for Wildlife Romania, Milvus Group, ММЕ Birdlife Hungary, Hortobagy NPD, Hungary, SPPN, Moldova, Agigea Ringing Station and Fundaţia Visul Luanei, Romania.

In memoriam to Michel Terrasse/LPO, VCF - a world-famous pioneer in vultures' re-introduction, conservation and knowledge-spreading, who was a great inspiration for most of us and passed away during the current manuscript writing in January 2023.

The publication of this research is financially supported by the French Embassy and the French Institute in Bulgaria.

## Supplementary Material

0FA1BA07-5774-557A-8A8E-952B6815254D 10.3897/BDJ.11.e100521.suppl1 Supplementary material 1 Table of all released Cinereous Vultures in Balkan Mountains in Bulgaria in 2018-2022 Data type released bird ID, origin, date of release, method of release, recoveries Brief description Details on marking, sex, origin, year of fledging, method, date and place of release and history of the Cinereous Vultures re-introduced and wild-fledged in the Balkan Mountains in 2018–2022. File: oo_818496.xlsx https://binary.pensoft.net/file/818496 Emilian Stoynov, Simeon Marin, George Stoyanov

## Figures and Tables

**Table 1.** The number of Cinereous Vultures released in the period 2018–2022 in Bulgaria per (sub)site, year and methods used – the numbers in brackets show the number of individuals released by hacking while the rest correspond to individuals released in aviaries.

Year	The Eastern Balkan Mountains		Total EBM	Vrachanski Balkan Nature Park	Total
	Sinite Kamani Nature Park	Kotlenska Planina SPA			
2018	-	(3)	(3)	-	**(3)**
2019	2 + (2)	9 + (2)	11 + (4)	-	**11 + (4)**
2020	-	-	-	10	**10**
2021	5	8	13	10	**23**
2022	(2)	8	8 + (2)	11	**19 + (2)**
Total	7 + (4)	25 + (2)	**32 + (9)**	**31**	**63 + (9)**

**Table 2.** The home range 95% and the core area 50% calculations in km^2^ of randomly selected individuals of Cinereous Vultures released (n = 3) and the wild caught one in EBM and released in VBNP (n = 4).

Ring code and name of the tracked Cinereous Vulture	# Days of tracking	Number of GPS fixes received	50% core area, km^2^	95% home range, km^2^
O5 – Montana	789	13,760	6.17	743.98
X3 - VCF Know-how	1,328	78,952	36.87	526.54
P2 - Varshets	885	52,987	13.84	409.36
K5 - Zlosten	911	50,611	22.70	421.48
P3 - Vrachanski Balkan	886	38,177	9.17	526.54
L5 - Balkan	1,356	30,197	43	600.08
P5 - Baraba	932	40,403	8.14	1,148.67
B1L - Regenerat	624	16,912	20.50	1,620.75

**Table 3.** Survival of the released Cinereous Vultures by site and period following the release. Note: the marked * figures are preliminary – the season is not completed at the reporting time and subsequent calculations are made on partial data. The figures in round brackets provide the rate of survival from the preceding period and the square brackets provide the rate of survivals in the respective period from all released birds in the batch.

	A. Number of birds released	B. Fledging	C. Acclimation	D. First autumn/ migration/ dispersal	E. First winter	F. First spring migration/ dispersal	G. One year after release	H. Two years after release	I. Three years after release	J. Four years after release
**Hacking**	**9**	**9**	**2**			**2**	**2**	**2**	**2**	**2**
		**(1.00)**	**(0.22)**			**(0.67)**	**(1.00)**	**(1.00)**	**(1.00)**	**(1.00)**
			[**0.22**]			[**0.22**]	[**0.22**]	[**0.22**]	[**0.22**]	[**0.22**]
Aviary EBM 2019	11	N/A	11	11	10	8	7	7	7	7
			(1.00)	(1.00)	(0.91)	(0.80)	(0.88)	(1.00)	(1.00)	(1.00)
				[1.00]	[0.91]	[0.73]	[0.64]	[0.64]	[0.64]	[0.64]
Aviary EBM 2021	13	N/A	7	7	4	4	4	3*	N/A	
			(0.54)	(1.00)	(0.57)	(1.00)	(1.00)	[0.75]		
				[0.54]	[0.31]	[0.31]	[0.31]	[0.23]		
Aviary EBM 2022	8	N/A	7	7	7*	N/A	N/A	N/A	N/A	N/A
			(0.88)	(1.00)	(1.00)					
				[0.88]	[0.88]					
Aviary VBNP 2020	10	N/A	7	7	7	7	7	6	N/A	N/A
			(0.70)	(1.00)	(1.00)	(1.00)	(1.00)	(0.86)		
				[0.70]	[0.70]	[0.70]	[0.70]	[0.60]		
Aviary VBNP 2021	10	N/A	9	9	9	9	9	8*	N/A	
			(0.90)	(1.00)	(1.00)	(1.00)	(1.00)	(0.89)		
				[0.90]	[0.90]	[0.90]	[0.90]	[0.80]		
Aviary VBNP 2022	11	N/A	11	11	9*	N/A	N/A	N/A	N/A	N/A
			(1.00)	(1.00)	(0.82)					
				[1.00]	[0.82]					
**Aviary Total**	**63**	**N/A**	**52**	**52**	46*(0.88)					
			**(0.83)**	**(1.00)**	[**0.73**]	**(0.93)**	**(0.96)**	**(0.89)**	**(1.00)**	**(1.00)**
				[**0.83**]		[**0.64**]	[**0.61**]	[**0.55**]	[**0.64**]	[**0.64**]
**TOTAL**	**72**	**(1.00)**	48*							
			-**0.67**			**(0.78)**	**(0.98)**	**(0.97)**	**(1.00)**	**(1.00)**
			[**0.67**]			[**0.57**]	[**0.55**]	[**0.49**]	[**0.45**]	[**0.45**]

**Table 4.** Mortality cases of released Cinereous Vultures in 2018–2022 by factor and period (during or beyond acclimation) and relative weight of the factor.

Mortality factor	During acclimation period	Beyond acclimation period	Total	Weight of the factor - % from all cases
Depredation	5	4	9	0.31
Gun fire shooting	4	1	5	0.17
Poisoning	1	4	5	0.17
Natural disease/ malfunction/ exhausting	3	-	3	0.10
Drowning	1	2	3	0.10
Electrocution/collision	-	3	3	0.10
Natural disasters	-	1	1	0.03
**Total**	**14**	**15**	**29**	1.00

**Table 5.** Fate of the released Cinereous Vultures in the Balkan Mountains in the period 2018–2022. * two of these birds are still immature and may return to the area of release, based on philopatry.

А. Number of dead individuals in the area of release (by reason)				B. Number of dead individuals outside the release area (by reason)				C. Breeds/sojourn anywhere out of the release area	D. Breeds/ sojourn in the release area	E. Unknown fate
depredation	shot	poison	other	depredation	shot	poison	other			
8	2	3	2	1	3	2	8			
15				14				4*	37	
29								41		2
**72**										

**Table 6.** Breeding performance and substrate of the nests of the re-introduced nuclei of the Cinereous Vulture in the two release sites in the Balkan Mountains during the period 2020–2022. * The number of nests is higher than the number of territorial pairs, as some pairs use more than one nest a season.

Year	Release site	# Territorial pairs (b)	# Breeding pairs/ laid eggs (c)	# Fledglings (d)	Breeding success (d/b)	Fledging success (d/c)	# Occupied nest	Substrate of the nest
2020	EBM	3	0	0	-	-	0	-
	VBNP	0	0	0	-	-	0	-
2021	EBM	3	2	1	0.33	0.5	3	Two artificial nests on oak, one natural on the ground
	VBNP	2	0	0	-	-	2	One natural on the ground (cliff) and one natural in Silver Pine.
2022	EBM	4	3	0	-	-	6*	Six artificial nests - three on oak, one on beech, one on sycamore and one in Austrian Pine.
	VBNP	3	1	1	0.33	1.0	2	One artificial on oak and one natural in Silver Pine.

**Table 7.** Breeding pairs, breeding attempts and breeding cycle data of the newly-established nuclei of the Cinereous Vulture in EBM and VBNP in 2021–2022.

Breeding pair – sex, name (year of hatching), site	Breeding season	Nest building	Copulation	Egg laying	Incubation period end	Hatching	Fledging	Independence of the chick
♂ Balkan (2017) x ♀ Kamchiya (2017), EBM	2021	February 2021	March 2021	17.03.2021	12.05.2021	12.05.2021	02.10.2021 (145^th^ day)	December 2021
♂ Kotel (2017) x ♀ VCF Know-how (2017), EBM	2021	February 2021	March 2021	20.03.2021	11.05.2021	none	-	-
♂ Zlosten (2017) x ♀ Marina (2017), EBM	2021	August 2021	-	-	-	-	-	-
♂ Varshets (2017) x ♀ Kotlya (2017), VBNP	2021	August 2021	-	-	-	-	-	-
♂ Vrachanski Balkan (2018) x ♀ Kutelka (2017), VBNP	2021	August 2021	-	-	-	-	-	-
-♂ Marto (2019) x ♀ Kamchiya (2017), EBM	2022	February 2022	-	12.03.2022	16.03.2022	none	-	-
♂ Kotel (2017) x ♀ VCF Know-how (2017), EBM	2022	February 2022	February 2022	22.02.2022	18.04.2022	-	-	-
♂ Zlosten (2017) x ♀ Montana (2018), EBM	2022	February 2021	February 2021	March 2021	May 2021	-	-	-
♂ Vrachanski Balkan (2018) x ♀ Kutelka (2017), VBNP	2022	August 2021	January 2022	06.02.2022	03.04.2022	03.04.2022	12.08.2022 (131^th^ day)	October 2022
♂ Varshets (2017) x ♀ Kotlya (2017), VBNP	2022	February 2022	-	-	-	-	-	-
♂ Baraba (2018) x ♀ Pateva (2020), VBNP	2022	August 2022	-	-	-	-	-	-
♂ Ozzy (2020) x ♀ Vaglen (2018), EBM	2022	August 2022	-	-	-	-	-	-
